# (*S*)-5-Oxo-*N*-phenyl­pyrrolidine-2-carboxamide

**DOI:** 10.1107/S160053681103786X

**Published:** 2011-09-30

**Authors:** Wei-Yan Qin, Bo Liu, Jing Ma, Hui-Juan Wang

**Affiliations:** a Key Laboratory of Green Chemical Technology, College of Heilongjiang Province, School of Chemistry and Environmental Engineering, Harbin University of Science and Technology, Harbin 150040, People’s Republic of China

## Abstract

The title compound, C_11_H_12_N_2_O_2_, shows an *S* configuration, in which the pyrrolidinone ring is twisted with respect to the phenyl plane, making a dihedral angle of 70.73 (7)°. In the crystal, mol­ecules are linked by N—H⋯O hydrogen bonds, building up a layer parallel to (001).

## Related literature

For the synthesis of the title compound, see Feng *et al.* (2010 ▶). For its chemical properties, including assignment of absolute structure, see: Brunel *et al.* (1999 ▶).
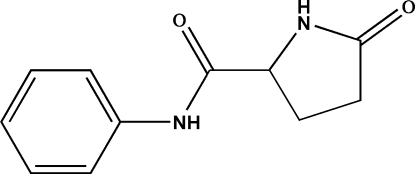


## Experimental

### 

#### Crystal data

C_11_H_12_N_2_O_2_
*M* *_r_* = 204.23
Monoclinic, 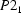

*a* = 4.919 (3) Å
*b* = 9.995 (7) Å
*c* = 10.382 (7) Å
β = 99.05 (3)°
*V* = 504.1 (6) Å^3^
*Z* = 2
Mo *K*α radiation
μ = 0.09 mm^−1^
*T* = 296 K
0.23 × 0.18 × 0.16 mm

#### Data collection

Rigaku R-AXIS RAPID diffractometer
Absorption correction: multi-scan *ABSCOR* (Higashi, 1995 ▶) *T*_min_ = 0.979, *T*_max_ = 0.985
3688 measured reflections
2184 independent reflections
1997 reflections with *I* > 2σ(*I*)
*R*_int_ = 0.014

#### Refinement

*R*[*F* ^2^ > 2σ(*F* ^2^)] = 0.032
*wR*(*F* ^2^) = 0.082
*S* = 1.05
2184 reflections
145 parameters
3 restraints
H atoms treated by a mixture of independent and constrained refinement
Δρ_max_ = 0.17 e Å^−3^
Δρ_min_ = −0.11 e Å^−3^

### 

Data collection: *RAPID-AUTO* (Rigaku, 1998 ▶); cell refinement: *RAPID-AUTO*; data reduction: *CrystalClear* (Rigaku/MSC, 2002 ▶); program(s) used to solve structure: *SHELXS97* (Sheldrick, 2008 ▶); program(s) used to refine structure: *SHELXL97* (Sheldrick, 2008 ▶); molecular graphics: *SHELXTL* (Sheldrick, 2008 ▶); software used to prepare material for publication: *SHELXL97*.

## Supplementary Material

Crystal structure: contains datablock(s) I, global. DOI: 10.1107/S160053681103786X/dn2719sup1.cif

Structure factors: contains datablock(s) I. DOI: 10.1107/S160053681103786X/dn2719Isup2.hkl

Supplementary material file. DOI: 10.1107/S160053681103786X/dn2719Isup3.cml

Additional supplementary materials:  crystallographic information; 3D view; checkCIF report

## Figures and Tables

**Table 1:** Hydrogen-bond geometry (Å, °)

*D*—H⋯*A*	*D*—H	H⋯*A*	*D*⋯*A*	*D*—H⋯*A*
N1—H11⋯O2^i^	0.89 (1)	1.98 (1)	2.869 (2)	172 (2)
N2—H12⋯O1^ii^	0.89 (1)	2.19 (1)	3.038 (2)	158 (2)

Symmetry codes: (i) 
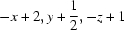
; (ii) 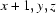.

**Fig. 1.**
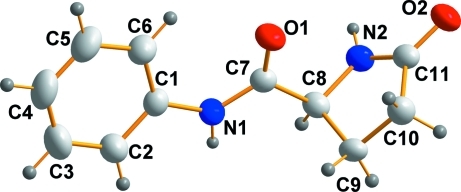
Molecular view of the title compound. Ellipsoids are drawn at the 50% probability level for non-H atoms.

**Fig. 2.**
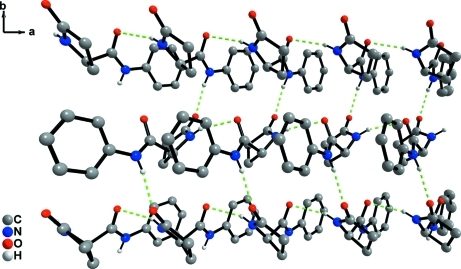
A partial packing view, showing hydrogen-bonding layer structure parallel to the (0 0 1) plane.

## supplementary crystallographic information

### Comment

The title compound is an intermediate in the synthesis of highly potent and selective insecticide (Feng *et al.*, 2010). Herein, we report its synthesis and crystal structure.

The pyrrolidinone ring is twisted with respect to phenyl plane with a dihedral angle of 70.73 (7) ° (Fig. 1).

Themolecules are linked by N—H···O hydrogen bonds into planar structure parallel to the (0 0 1) plane (Fig. 2, Table 1).

### Experimental

The title compound was synthesized as the reference method (Feng *et al.*, 2010; Brunel *et al.*, 1999): a mixture of L-glutamic acid (3 g) and aniline (18 mL) was stirred at 195-200 °C. After 30 min, the mixture became clear, and the water formed was removed by azeotropic distillation. Stirring was maintained for 4 h. Excess of aniline was then recovered at 60-70 °C under reduced pressure distillation. The hot oily residue was swirled with acetone (25 mL) to lead to the formation of a brown solid, which was collected by filtration and dissolved in hot methanol (40 mL). The solution was slowly cooled to room temperature to afford crystalline optically pure *(S)*-*N*-phenylpyrrolidine-2-carboxamide as white crystals in 85% with the specific rotation about [α]^20^_D_ + 18.0 (c 1.0, MeOH, 24 °C).

### Refinement

H atoms bound to C atoms were placed in calculated positions and treated as riding on their parent atoms, with C—H = 0.93 Å (aromatic); C—H = 0.97 Å (methylene), and C—H = 0.98 Å (methine), and with *U*_iso_(H) = 1.2*U*_eq_(C), while N-bound H atoms were found from difference Fourier and were refined using restraints [ N—H = 0.90 (1)Å].

### Crystal data

**Table:**

C_11_H_12_N_2_O_2_	*F*(000) = 216
*M**_r_* = 204.23	*D*_x_ = 1.345 Mg m^−^^3^
Monoclinic, *P*2_1_	Mo *K*α radiation, λ = 0.71073 Å
Hall symbol: P 2yb	Cell parameters from 1906 reflections
*a* = 4.919 (3) Å	θ = 2.9–28.3°
*b* = 9.995 (7) Å	µ = 0.09 mm^−^^1^
*c* = 10.382 (7) Å	*T* = 296 K
β = 99.05 (3)°	Block, yellow
*V* = 504.1 (6) Å^3^	0.23 × 0.18 × 0.16 mm
*Z* = 2	

### Data collection

**Table:**

Rigaku R-AXIS RAPID diffractometer	2184 independent reflections
Radiation source: fine-focus sealed tube	1997 reflections with *I* > 2σ(*I*)
graphite	*R*_int_ = 0.014
ω scans	θ_max_ = 28.4°, θ_min_ = 2.0°
Absorption correction: multi-scan *ABSCOR* (Higashi, 1995)	*h* = −4→6
*T*_min_ = 0.979, *T*_max_ = 0.985	*k* = −12→13
3688 measured reflections	*l* = −13→11

### Refinement

**Table:**

Refinement on *F*^2^	Secondary atom site location: difference Fourier map
Least-squares matrix: full	Hydrogen site location: inferred from neighbouring sites
*R*[*F*^2^ > 2σ(*F*^2^)] = 0.032	H atoms treated by a mixture of independent and constrained refinement
*wR*(*F*^2^) = 0.082	*w* = 1/[σ^2^(*F*_o_^2^) + (0.0429*P*)^2^ + 0.035*P*] where *P* = (*F*_o_^2^ + 2*F*_c_^2^)/3
*S* = 1.05	(Δ/σ)_max_ < 0.001
2184 reflections	Δρ_max_ = 0.17 e Å^−^^3^
145 parameters	Δρ_min_ = −0.11 e Å^−^^3^
3 restraints	Extinction correction: *SHELXL97* (Sheldrick, 2008)
Primary atom site location: structure-invariant direct methods	Extinction coefficient: 0.024 (6)

### Special details

**Table:**

Geometry. All esds (except the esd in the dihedral angle between two l.s. planes) are estimated using the full covariance matrix. The cell esds are taken into account individually in the estimation of esds in distances, angles and torsion angles; correlations between esds in cell parameters are only used when they are defined by crystal symmetry. An approximate (isotropic) treatment of cell esds is used for estimating esds involving l.s. planes.
Refinement. Refinement of F^2^ against ALL reflections. The weighted R-factor wR and goodness of fit S are based on F^2^, conventional R-factors R are based on F, with F set to zero for negative F^2^. The threshold expression of F^2^ > 2sigma(F^2^) is used only for calculating R-factors(gt) etc. and is not relevant to the choice of reflections for refinement. R-factors based on F^2^ are statistically about twice as large as those based on F, and R- factors based on ALL data will be even larger.

### Fractional atomic coordinates and isotropic or equivalent isotropic displacement parameters (Å^2^)

**Table:**

	*x*	*y*	*z*	*U*_iso_*/*U*_eq_	
C1	0.5338 (3)	0.92196 (15)	0.78902 (13)	0.0367 (3)	
C2	0.4415 (4)	1.02616 (18)	0.85717 (15)	0.0487 (4)	
H2	0.4934	1.1134	0.8416	0.058*	
C3	0.2721 (4)	1.0014 (2)	0.94858 (18)	0.0615 (5)	
H3	0.2118	1.0721	0.9951	0.074*	
C4	0.1922 (4)	0.8728 (2)	0.97111 (16)	0.0602 (5)	
H4	0.0767	0.8561	1.0321	0.072*	
C5	0.2838 (4)	0.7705 (2)	0.90327 (17)	0.0586 (5)	
H5	0.2297	0.6835	0.9185	0.070*	
C6	0.4553 (4)	0.79267 (18)	0.81216 (16)	0.0483 (4)	
H6	0.5171	0.7214	0.7670	0.058*	
C7	0.7855 (2)	0.87730 (14)	0.60470 (12)	0.0337 (3)	
C8	0.9689 (3)	0.94686 (15)	0.51984 (13)	0.0360 (3)	
H8	1.0871	1.0137	0.5700	0.043*	
C9	0.7915 (3)	1.01089 (16)	0.40036 (14)	0.0413 (3)	
H9A	0.6115	1.0355	0.4197	0.050*	
H9B	0.8801	1.0898	0.3719	0.050*	
C10	0.7685 (3)	0.90159 (17)	0.29767 (14)	0.0449 (4)	
H10A	0.6000	0.8508	0.2960	0.054*	
H10B	0.7722	0.9391	0.2118	0.054*	
C11	1.0170 (3)	0.81512 (14)	0.33982 (14)	0.0373 (3)	
N1	0.7110 (2)	0.95458 (13)	0.69845 (11)	0.0385 (3)	
N2	1.1299 (2)	0.85149 (13)	0.46021 (11)	0.0388 (3)	
O1	0.7047 (2)	0.76321 (11)	0.58091 (10)	0.0443 (3)	
O2	1.1033 (3)	0.72678 (12)	0.27523 (12)	0.0538 (3)	
H11	0.784 (3)	1.0363 (11)	0.7055 (16)	0.041 (4)*	
H12	1.273 (3)	0.8084 (17)	0.5056 (15)	0.050 (5)*	

### Atomic displacement parameters (Å^2^)

**Table:**

	*U*^11^	*U*^22^	*U*^33^	*U*^12^	*U*^13^	*U*^23^
C1	0.0352 (6)	0.0417 (8)	0.0334 (6)	−0.0014 (6)	0.0056 (5)	0.0025 (5)
C2	0.0548 (9)	0.0447 (9)	0.0494 (8)	0.0007 (7)	0.0166 (7)	−0.0014 (7)
C3	0.0635 (10)	0.0709 (13)	0.0555 (10)	0.0069 (10)	0.0262 (8)	−0.0052 (9)
C4	0.0541 (9)	0.0852 (15)	0.0446 (8)	−0.0073 (10)	0.0178 (7)	0.0076 (10)
C5	0.0620 (10)	0.0636 (12)	0.0523 (9)	−0.0153 (9)	0.0157 (8)	0.0113 (8)
C6	0.0559 (9)	0.0435 (9)	0.0477 (8)	−0.0071 (7)	0.0146 (7)	0.0024 (7)
C7	0.0301 (5)	0.0332 (7)	0.0371 (6)	0.0013 (5)	0.0034 (5)	0.0012 (5)
C8	0.0322 (6)	0.0332 (7)	0.0437 (7)	−0.0036 (5)	0.0091 (5)	−0.0042 (6)
C9	0.0433 (7)	0.0323 (7)	0.0505 (8)	0.0080 (6)	0.0147 (6)	0.0060 (6)
C10	0.0453 (7)	0.0479 (9)	0.0415 (7)	0.0079 (7)	0.0067 (6)	0.0015 (6)
C11	0.0379 (7)	0.0317 (7)	0.0452 (7)	0.0000 (5)	0.0151 (6)	0.0031 (6)
N1	0.0439 (6)	0.0332 (7)	0.0403 (6)	−0.0069 (5)	0.0127 (5)	−0.0030 (5)
N2	0.0295 (5)	0.0416 (7)	0.0461 (6)	0.0063 (5)	0.0086 (4)	0.0022 (5)
O1	0.0458 (5)	0.0333 (5)	0.0561 (6)	−0.0059 (4)	0.0152 (5)	−0.0061 (4)
O2	0.0670 (7)	0.0397 (7)	0.0591 (7)	0.0090 (5)	0.0236 (6)	−0.0055 (5)

### Geometric parameters (Å, °)

**Table:**

C1—C2	1.376 (2)	C7—C8	1.524 (2)
C1—C6	1.381 (2)	C8—N2	1.4400 (19)
C1—N1	1.4170 (19)	C8—C9	1.539 (2)
C2—C3	1.380 (3)	C8—H8	0.9800
C2—H2	0.9300	C9—C10	1.518 (2)
C3—C4	1.375 (3)	C9—H9A	0.9700
C3—H3	0.9300	C9—H9B	0.9700
C4—C5	1.358 (3)	C10—C11	1.505 (2)
C4—H4	0.9300	C10—H10A	0.9700
C5—C6	1.381 (2)	C10—H10B	0.9700
C5—H5	0.9300	C11—O2	1.2244 (18)
C6—H6	0.9300	C11—N2	1.336 (2)
C7—O1	1.2201 (19)	N1—H11	0.891 (9)
C7—N1	1.3380 (19)	N2—H12	0.893 (9)
			
C2—C1—C6	119.69 (14)	N2—C8—H8	111.0
C2—C1—N1	117.01 (14)	C7—C8—H8	111.0
C6—C1—N1	123.30 (13)	C9—C8—H8	111.0
C1—C2—C3	120.12 (18)	C10—C9—C8	103.69 (13)
C1—C2—H2	119.9	C10—C9—H9A	111.0
C3—C2—H2	119.9	C8—C9—H9A	111.0
C4—C3—C2	120.27 (18)	C10—C9—H9B	111.0
C4—C3—H3	119.9	C8—C9—H9B	111.0
C2—C3—H3	119.9	H9A—C9—H9B	109.0
C5—C4—C3	119.24 (16)	C11—C10—C9	104.00 (13)
C5—C4—H4	120.4	C11—C10—H10A	111.0
C3—C4—H4	120.4	C9—C10—H10A	111.0
C4—C5—C6	121.53 (19)	C11—C10—H10B	111.0
C4—C5—H5	119.2	C9—C10—H10B	111.0
C6—C5—H5	119.2	H10A—C10—H10B	109.0
C1—C6—C5	119.15 (17)	O2—C11—N2	125.43 (14)
C1—C6—H6	120.4	O2—C11—C10	126.21 (14)
C5—C6—H6	120.4	N2—C11—C10	108.36 (13)
O1—C7—N1	124.68 (13)	C7—N1—C1	128.11 (13)
O1—C7—C8	120.86 (13)	C7—N1—H11	115.8 (12)
N1—C7—C8	114.32 (12)	C1—N1—H11	116.1 (11)
N2—C8—C7	111.24 (12)	C11—N2—C8	113.99 (11)
N2—C8—C9	102.08 (12)	C11—N2—H12	122.6 (12)
C7—C8—C9	110.08 (12)	C8—N2—H12	122.2 (12)

### Hydrogen-bond geometry (Å, °)

**Table:**

*D*—H···*A*	*D*—H	H···*A*	*D*···*A*	*D*—H···*A*
N1—H11···O2^i^	0.89 (1)	1.98 (1)	2.869 (2)	172.(2)
N2—H12···O1^ii^	0.89 (1)	2.19 (1)	3.038 (2)	158.(2)

Symmetry codes: (i) −*x*+2, *y*+1/2, −*z*+1; (ii) *x*+1, *y*, *z*.
